# Supplementary material for: Genome-wide classification of tumor-derived reads from bulk long-read sequencing
Source: bioRxiv. 2026 Mar 5:2026.03.03.709085. Preprint. [Version 1] doi: 10.64898/2026.03.03.709085 (PMC12991090; doi:10.64898/2026.03.03.709085)
Supplement: 1 [file NIHPP2026.03.03.709085v1-supplement-1.pdf]

bioRxiv preprint doi: https://doi.org/10.64898/2026.03.03.709085; this version posted March 5, 2026. The copyright holder for this preprint (which was not certified by peer review) is the author/funder, who has granted bioRxiv a license to display the preprint in perpetuity. It is made available under aCC-BY 4.0 International license.

Supplementary Figures

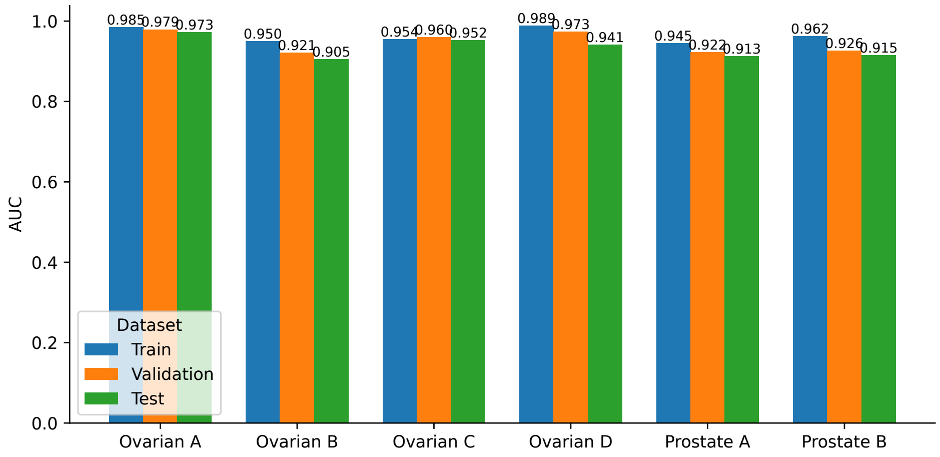

Figure S1: Area under the receiver operator curve results for the train, validation and test set datasets for the transformer model on the four ovarian and two prostate samples.

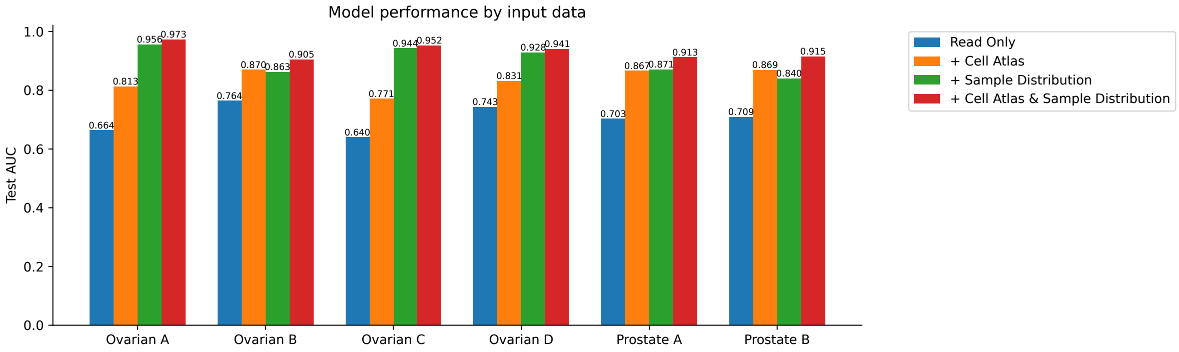

Figure S2: AUC results on the test datasets with different input features for classification. Results displayed for training on read level methylation alone, incorporating the CpG sample distributions and cell type methylation reference atlases separately and together.

bioRxiv preprint doi: https://doi.org/10.64898/2026.03.03.709085; this version posted March 5, 2026. The copyright holder for this preprint (which was not certified by peer review) is the author/funder, who has granted bioRxiv a license to display the preprint in perpetuity. It is made available under aCC-BY 4.0 International license.

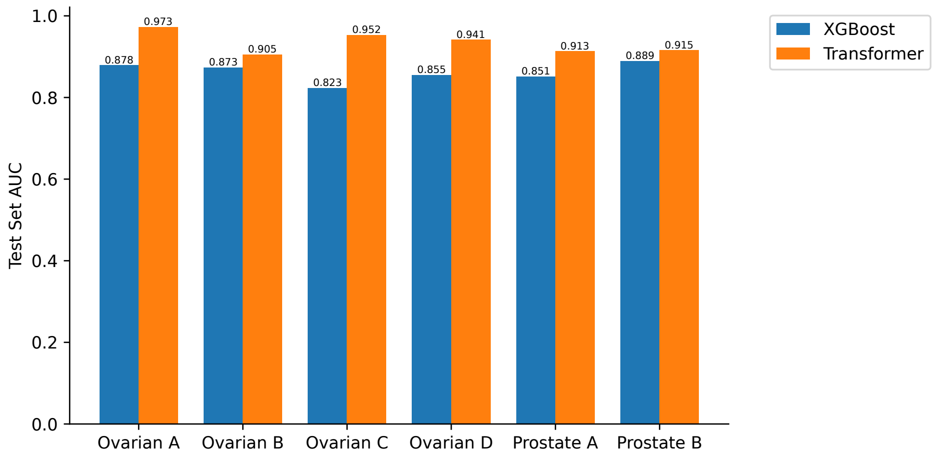

Figure S3: Area under the receiver operator curve results on test set datasets for the transformer and XGBoost models trained and evaluated on the same sample for the four ovarian and two prostate samples.

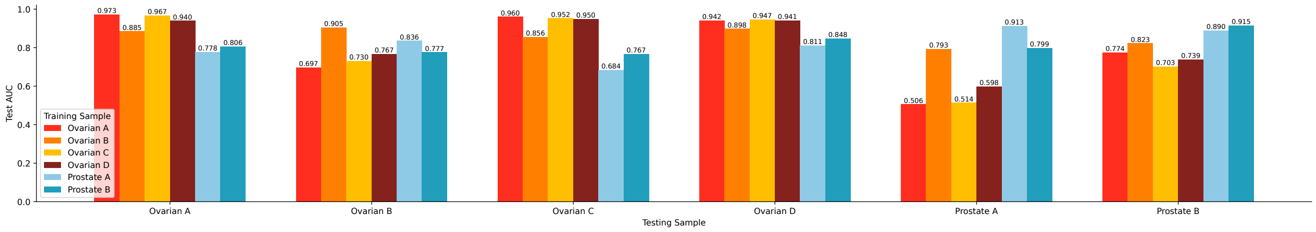

Figure S4: Area under the receiver operator curve results for the transformer model trained on the read data from one of the samples and applied to test set distribution of labeled reads from all samples separately.

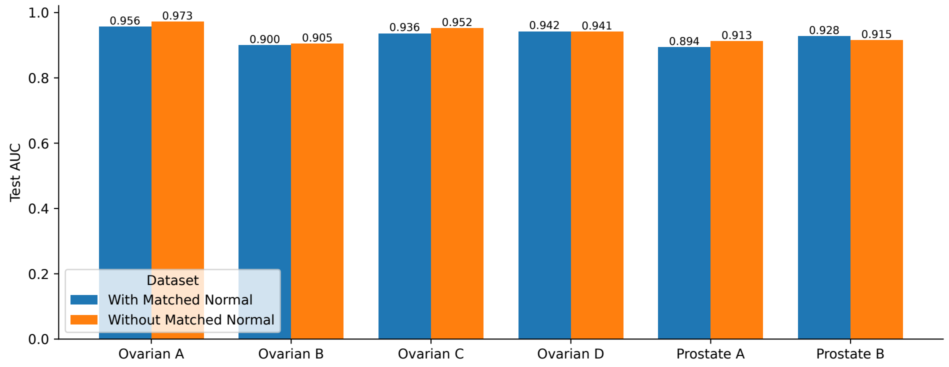

Figure S5: Area under the receiver operator curve results on test set datasets for transformer models with and without matched normal reads added to the training set. No matched normal reads were added to the test set.

bioRxiv preprint doi: https://doi.org/10.64898/2026.03.03.709085; this version posted March 5, 2026. The copyright holder for this preprint (which was not certified by peer review) is the author/funder, who has granted bioRxiv a license to display the preprint in perpetuity. It is made available under aCC-BY 4.0 International license.

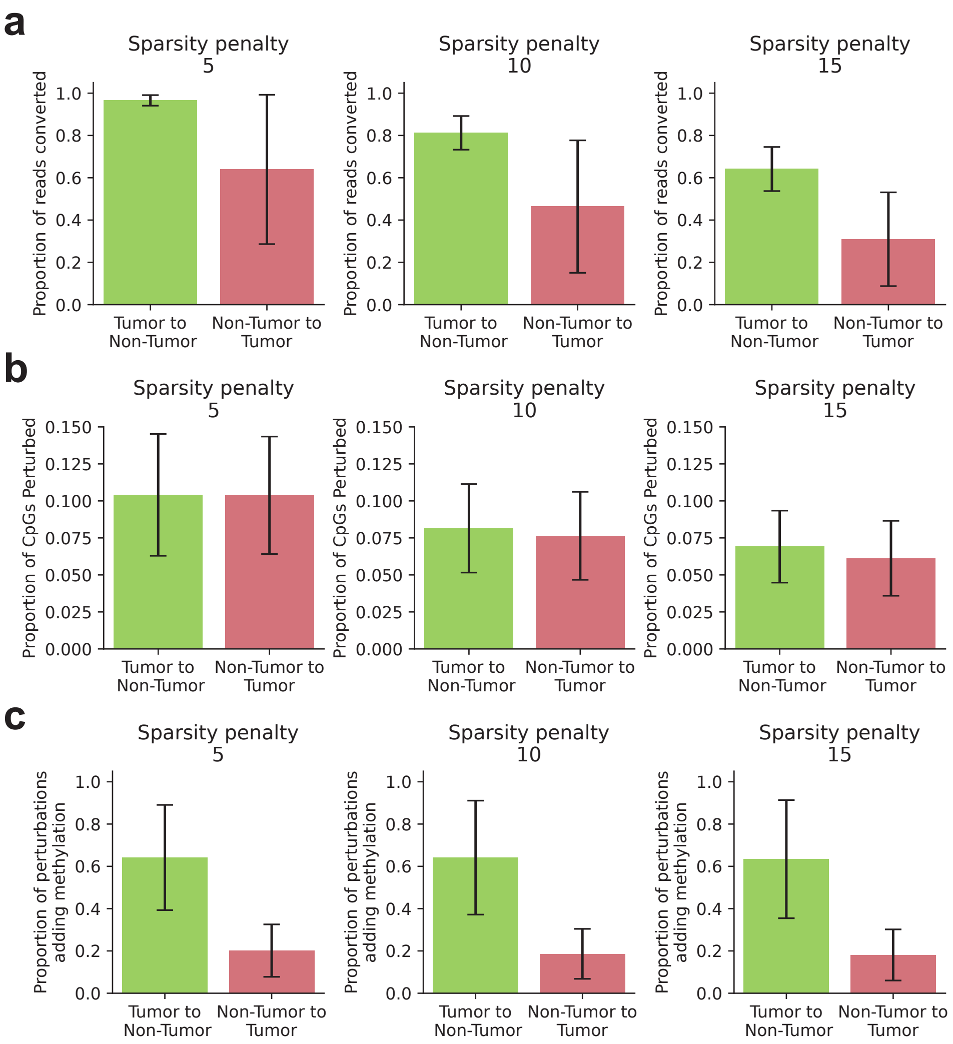

Figure S6: **a–c.** Error bars indicate standard deviation across samples. **a.** The proportion of reads successfully converted by CpG methylation perturbations for different L_0 sparsity penalty values. **b.** The proportion of perturbed CpG sites in reads with successfully converted classifications for different L_0 sparsity penalty values. **c.** The proportion of perturbed CpG sites where the methylation probability was increased in reads with successfully converted classifications for different L_0 sparsity penalty values.

bioRxiv preprint doi: https://doi.org/10.64898/2026.03.03.709085; this version posted March 5, 2026. The copyright holder for this preprint (which was not certified by peer review) is the author/funder, who has granted bioRxiv a license to display the preprint in perpetuity. It is made available under aCC-BY 4.0 International license.

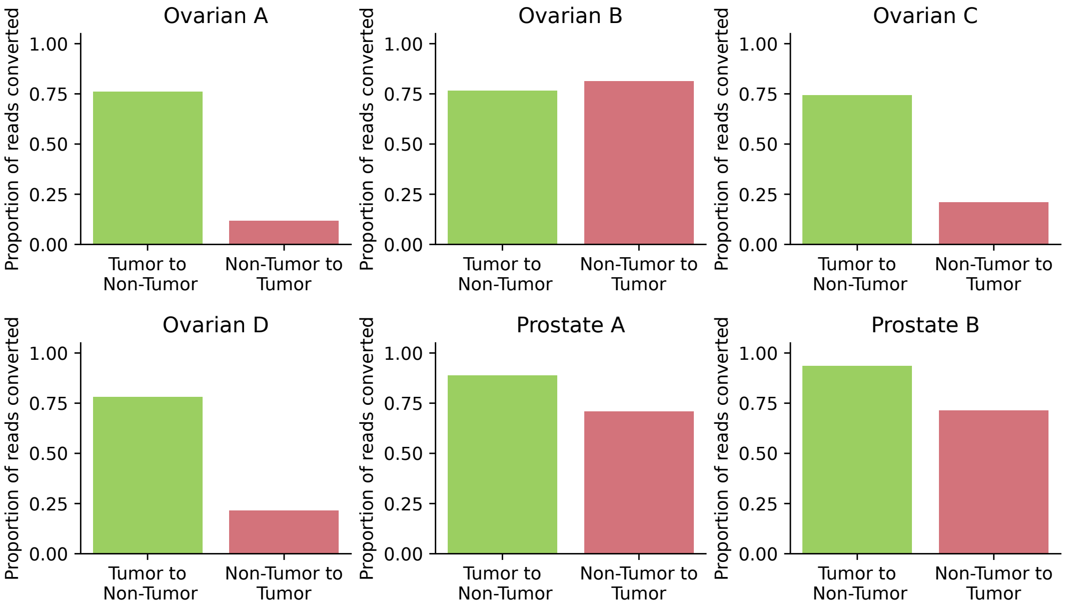

Figure S7: The proportion of reads successfully converted by CpG methylation perturbations split by the samples in the cohort.

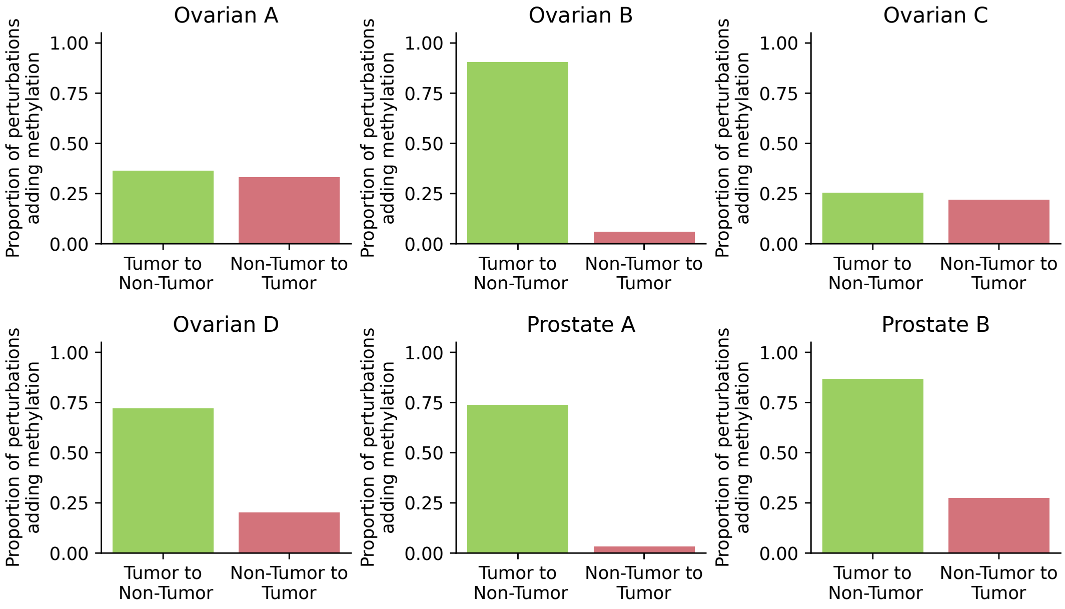

Figure S8: The proportion of perturbed CpG sites where the methylation probability was increased in reads with successfully converted classifications split by the samples in the cohort.

bioRxiv preprint doi: https://doi.org/10.64898/2026.03.03.709085; this version posted March 5, 2026. The copyright holder for this preprint (which was not certified by peer review) is the author/funder, who has granted bioRxiv a license to display the preprint in perpetuity. It is made available under aCC-BY 4.0 International license.

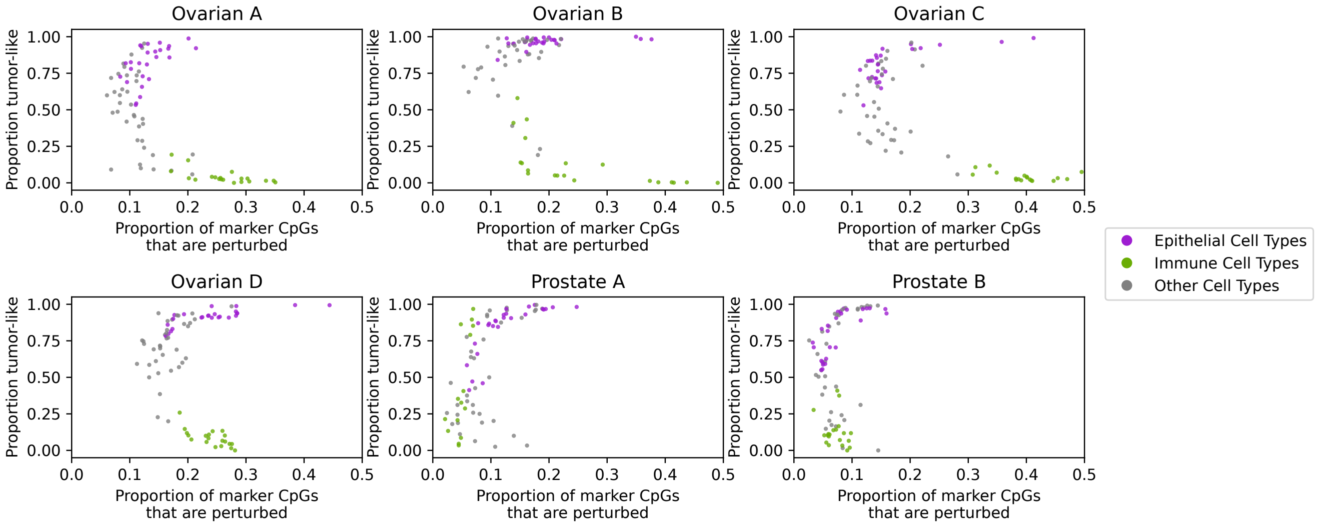

Figure S9: The proportion of marker CpGs for a given cell type that were perturbed in reads with successful perturbations against the proportion of perturbations for each marker CpG where the marker was associated with increased tumor origin probability for a read.

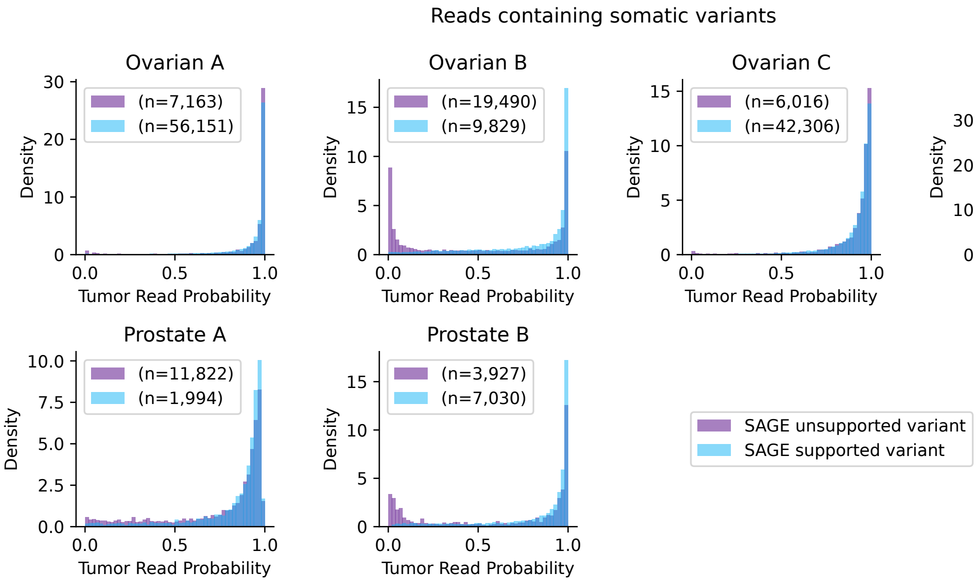

Figure S10: Tumor read probabilities for each sample for reads containing somatic variants identified by DeepSomatic from long read sequencing. Distributions split by whether each variant was identified as a high quality variant by SAGE using short read sequencing.

bioRxiv preprint doi: https://doi.org/10.64898/2026.03.03.709085; this version posted March 5, 2026. The copyright holder for this preprint (which was not certified by peer review) is the author/funder, who has granted bioRxiv a license to display the preprint in perpetuity. It is made available under aCC-BY 4.0 International license.

Supplementary Tables

Sample ID	Training Tumor Reads	Training Non-Tumor Reads
Ovarian A	97842 (1.0%)	48975 (0.5%)
Ovarian B	29013 (0.2%)	149924 (1.2%)
Ovarian C	80910 (0.7%)	42110 (0.4%)
Ovarian D	34221 (0.3%)	63111 (0.5%)
Prostate A	18697 (0.2%)	51204 (0.5%)
Prostate B	22215 (0.2%)	149547 (1.5%)

Sample ID	Testing Tumor Reads	Testing Non-Tumor Reads
Ovarian A	6129 (0.1%)	8927 (0.1%)
Ovarian B	3051 (0.0%)	3621 (0.0%)
Ovarian C	6841 (0.1%)	7219 (0.1%)
Ovarian D	2615 (0.0%)	53583 (0.4%)
Prostate A	2196 (0.0%)	2582 (0.0%)
Prostate B	2631 (0.0%)	2179 (0.0%)

Sample ID	Validation Tumor Reads	Validation Non-Tumor Reads
Ovarian A	6496 (0.1%)	7178 (0.1%)
Ovarian B	2937 (0.0%)	3207 (0.0%)
Ovarian C	6504 (0.1%)	10685 (0.1%)
Ovarian D	4073 (0.0%)	3609 (0.0%)
Prostate A	2027 (0.0%)	1980 (0.0%)
Prostate B	2211 (0.0%)	1700 (0.0%)

Sample ID	Unlabeled Reads
Ovarian A	9830358 (98.2%)
Ovarian B	12409953 (98.5%)
Ovarian C	10968121 (98.6%)
Ovarian D	12115216 (98.7%)
Prostate A	10511605 (99.3%)
Prostate B	9836171 (98.2%)

Supplementary Table 1: Classification counts for ground truth training data for the five samples in our cohort. The remaining reads are the reads for each sample where no ground truth origin label could be obtained. Percentages are expressed as a fraction of the total bulk tumor reads for the sample.

bioRxiv preprint doi: https://doi.org/10.64898/2026.03.03.709085; this version posted March 5, 2026. The copyright holder for this preprint (which was not certified by peer review) is the author/funder, who has granted bioRxiv a license to display the preprint in perpetuity. It is made available under aCC-BY 4.0 International license.

Ovarian A

Actual	Predicted Non-Tumor	Predicted Tumor
Non-Tumor	44373	4602
Tumor	3178	94664

Train Dataset

Actual	Predicted Non-Tumor	Predicted Tumor
Non-Tumor	6578	600
Tumor	372	6124

Validation Dataset

Actual	Predicted Non-Tumor	Predicted Tumor
Non-Tumor	7995	932
Tumor	364	5765

Test Dataset

Supplementary Table 2: Confusion matrix for the labeled training, testing and validation datasets for the model trained and evaluated on sample ovarian A. A probability threshold of 0.5 was used for tumor read classification.

Ovarian B

Actual	Predicted Non-Tumor	Predicted Tumor
Non-Tumor	131583	18341
Tumor	3731	25282

Train Dataset

Actual	Predicted Non-Tumor	Predicted Tumor
Non-Tumor	2673	534
Tumor	479	2458

Validation Dataset

Actual	Predicted Non-Tumor	Predicted Tumor
Non-Tumor	2854	767
Tumor	522	2529

Test Dataset

Supplementary Table 3: Confusion matrix for the labeled training, testing and validation datasets for the model trained and evaluated on sample ovarian B. A probability threshold of 0.5 was used for tumor read classification.

bioRxiv preprint doi: https://doi.org/10.64898/2026.03.03.709085; this version posted March 5, 2026. The copyright holder for this preprint (which was not certified by peer review) is the author/funder, who has granted bioRxiv a license to display the preprint in perpetuity. It is made available under aCC-BY 4.0 International license.

Ovarian C

Actual	Predicted Non-Tumor	Predicted Tumor
Non-Tumor	32801	9309
Tumor	2568	78342

Train Dataset

Actual	Predicted Non-Tumor	Predicted Tumor
Non-Tumor	8667	2018
Tumor	232	6272

Validation Dataset

Actual	Predicted Non-Tumor	Predicted Tumor
Non-Tumor	5677	1542
Tumor	235	6606

Test Dataset

Supplementary Table 4: Confusion matrix for the labeled training, testing and validation datasets for the model trained and evaluated on sample ovarian C. A probability threshold of 0.5 was used for tumor read classification.

Ovarian D

Actual	Predicted Non-Tumor	Predicted Tumor
Non-Tumor	58830	4281
Tumor	1216	33005

Train Dataset

Actual	Predicted Non-Tumor	Predicted Tumor
Non-Tumor	3212	397
Tumor	214	3859

Validation Dataset

Actual	Predicted Non-Tumor	Predicted Tumor
Non-Tumor	46282	7301
Tumor	404	2211

Test Dataset

Supplementary Table 5: Confusion matrix for the labeled training, testing and validation datasets for the model trained and evaluated on sample ovarian D. A probability threshold of 0.5 was used for tumor read classification.

bioRxiv preprint doi: https://doi.org/10.64898/2026.03.03.709085; this version posted March 5, 2026. The copyright holder for this preprint (which was not certified by peer review) is the author/funder, who has granted bioRxiv a license to display the preprint in perpetuity. It is made available under aCC-BY 4.0 International license.

Prostate A

**Actual**	**Predicted** Non-Tumor	Tumor
Non-Tumor	41305	9899
Tumor	1349	17348

Train Dataset

**Actual**	**Predicted** Non-Tumor	Tumor
Non-Tumor	1551	429
Tumor	192	1835

Validation Dataset

**Actual**	**Predicted** Non-Tumor	Tumor
Non-Tumor	2013	569
Tumor	241	1955

Test Dataset

Supplementary Table 6: Confusion matrix for the labeled training, testing and validation datasets for the model trained and evaluated on sample prostate A. A probability threshold of 0.5 was used for tumor read classification.

Prostate B

**Actual**	**Predicted** Non-Tumor	Tumor
Non-Tumor	133085	16462
Tumor	2152	20063

Train Dataset

**Actual**	**Predicted** Non-Tumor	Tumor
Non-Tumor	1419	281
Tumor	290	1921

Validation Dataset

**Actual**	**Predicted** Non-Tumor	Tumor
Non-Tumor	1813	366
Tumor	421	2210

Test Dataset

Supplementary Table 7: Confusion matrix for the labeled training, testing and validation datasets for the model trained and evaluated on sample prostate B. A probability threshold of 0.5 was used for tumor read classification.
